# Biomimetic Targeted Drug Delivery for Liver Failure in Abdominal Sepsis: Focus on Autologous Erythrocyte Ghosts

**DOI:** 10.3390/ijms27114978

**Published:** 2026-05-30

**Authors:** Kulzhan Berikkhanova, Isah Inuwa, Erlan Taigulov, Saken Kozhakhmetov, Nurzhan Bikhanov, Ardak Omarbekov, Gulsara Berikkhanova, Yessenhan Sultan, Abdulrahman Garba Jibo, Saniya Abdrakhmanova, Zhannat Zhakiyanova, Gulyash Tanysheva, Zhaxybay Zhumadilov

**Affiliations:** 1 National Laboratory Astana, Nazarbayev University, Astana 010000, Kazakhstan; 2 University Medical Center, Nazarbayev University, Astana 010000, Kazakhstan; 3 Professor G.V. Tsoi Scientific and Educational Center of Surgery, Astana Medical University, Astana 010000, Kazakhstan; 4 School of Medicine, Nazarbayev University, Astana 010000, Kazakhstan; 5 Department of Higher Mathematics, Faculty of Computer Systems and Professional Education, Saken Seifullin Kazakh Agrotechnical University, Astana 010000, Kazakhstan; 6 Medical Center “Sultan-Medicus”, Almaty 050000, Kazakhstan; 7 Faculty of Pharmaceutical Sciences, Chulalongkorn University, Bangkok 10330, Thailand; 8 “Scientific and Production Center for Transfusiology” of the Ministry of Health of the Republic of Kazakhstan, Astana 010000, Kazakhstan; 9 Department of Neurology, Ophthalmology and Otorhinolaryngology, Department of Obstetrics and Gynecology named after A.A. Kozbagarov, Semey Medical University, Semey 071400, Kazakhstan

**Keywords:** sepsis-induced liver failure, liver-targeted drug delivery, erythrocyte ghosts, biomimetic nanocarriers, septic pathophysiology

## Abstract

Sepsis-induced liver failure remains a serious and often under-recognized complication of abdominal sepsis. Clinical reports suggest that liver dysfunction develops in a substantial proportion of these patients, and once failure ensues, mortality rises sharply. Despite progress in antimicrobial therapy and critical care support, there is still no therapy that directly halts or reliably reverses septic liver injury. Systemic drug administration frequently underperforms in this setting. Hepatic drug accumulation becomes unpredictable, pharmacokinetics shift, and immune dysregulation further complicates therapeutic control. Nanotechnology-based delivery systems have attempted to address these shortcomings by improving drug stability and circulation time. Yet their behavior under septic conditions remains inconsistent. This inconsistency may reflect a deeper issue: most carriers are engineered under relatively stable physiological assumptions that do not hold during systemic inflammation. Biomimetic platforms, particularly those derived from erythrocyte membranes, offer a different conceptual entry point. Rather than merely evading immune recognition, erythrocyte-based systems interact naturally with hepatic clearance pathways. During sepsis, erythrocyte turnover appears to accelerate, and macrophage-mediated clearance in the liver intensifies. This shift, while pathologic, may present a therapeutic opportunity. In this review, we examine current liver-targeted delivery strategies for sepsis-induced liver failure and critically assess the underexplored role of erythrocyte ghost-based systems. We discuss how sepsis-specific pathophysiological changes reshape carrier biodistribution, identify translational constraints, and propose design considerations for inflammation-adaptive biomimetic platforms. By reconsidering hepatic clearance not solely as a pharmacokinetic barrier but as a potential delivery route, we outline a disease-aligned approach to nanomedicine design in septic organ failure.

## 1. Introduction

As established by the Third International Consensus Definitions for Sepsis and Septic Shock (Sepsis-3), sepsis is defined as a life-threatening organ dysfunction caused by a dysregulated host response to infection [1]. This physiological deterioration is clinically assessed using the Sequential Organ Failure Assessment (SOFA) score, whereby an acute rise of 2 points or more indicates significant organ impairment. Epidemiologically, sepsis continues to impose a substantial global burden. Estimates from 1990 to 2017 indicate nearly 49 million incident cases and approximately 11 million related deaths worldwide, accounting for close to one-fifth of all global mortality [2]. The impact is particularly pronounced in low- and middle-income settings, where limited access to advanced organ support worsens outcomes [3]. Even in well-resourced systems, once sepsis progresses to multiple organ dysfunction syndrome (MODS), prognosis remains guarded [4]. Among affected organs, the liver occupies a paradoxical position. It is both central to host defense and highly susceptible to injury. As the primary site of detoxification, metabolic regulation, and immune surveillance, it is continuously exposed to circulating pathogens, endotoxins, and inflammatory mediators [5]. In abdominal sepsis, this exposure becomes sustained and amplified. Consequently, sepsis-induced liver failure is consistently associated with increased mortality and is often considered an independent predictor of poor outcomes [6,7].

The pathogenesis of liver failure in sepsis is complex and multifactorial, involving dysregulated immune responses, microcirculatory disturbances, mitochondrial dysfunction, and excessive cytokine release [8]. These processes do more than damage hepatocytes. They also alter drug handling–reshaping pharmacokinetics and biodistribution in ways that complicate therapy [9]. Capillary leak, endothelial dysfunction, hypoalbuminemia, and macrophage activation together create a dynamic and, at times, hostile delivery environment [10]. Under such conditions, drugs administered systemically may fail to reach therapeutic hepatic concentrations, while off-target exposure increases toxicity risk [11]. These limitations highlight a fundamental gap between current therapeutic approaches and the biological realities of septic liver injury.

To address these challenges, liver-targeted drug delivery has emerged as a strategy to improve therapeutic precision while minimizing systemic exposure [12]. Advances in nanotechnology have enabled the development of liposomes, polymeric nanoparticles, and hybrid nanocarriers that demonstrate improved drug stability and hepatic accumulation in stable disease models [13,14]. However, most of these nanomaterial-based systems exhibit poor cellular uptake and low biocompatibility, which leads to suboptimal drug efficacy [15,16]. Moreover, these systems were often optimized under relatively stable physiological conditions and rely on assumptions, such as preserved vascular integrity and stable receptor expression, which do not hold true during sepsis. As a result, their effectiveness and translational success in sepsis-induced liver failure have been limited [17,18]. Biomimetic delivery systems introduce a different logic. Instead of shielding synthetic carriers from biology, they draw directly from it [19]. Erythrocytes, macrophages, platelets, leukocytes, and even tumor cells have been explored as source materials for biologically integrated carriers [20,21]. Among these, erythrocyte-derived systems are particularly compelling. They offer intrinsic biocompatibility, long circulation half-life, and well-characterized interactions with the mononuclear phagocyte system [22,23]. What is often described as a limitation—their tendency to accumulate in the liver and spleen—may, in septic liver injury, become an advantage.

Autologous erythrocyte ghosts extend this idea further by leveraging endogenous clearance pathways. Under physiological conditions, erythrocytes express surface proteins such as CD47, which interact with inhibitory receptor signal regulatory protein alpha (SIRPα) on macrophages to transmit a “self” signal and delay phagocytosis [24,25]. Yet, in sepsis, erythrocyte turnover appears to accelerate, and hepatic macrophages become highly active. In this context, erythrocyte ghosts may exploit CD47-mediated signaling to promote liver-specific clearance, which enhances their targeting to macrophages in the liver [26,27,28]. This mechanism can be therapeutically beneficial in liver-targeted drug delivery systems but could exacerbate pathological damage if not carefully controlled. Preclinical studies have demonstrated this principle using red blood cell-derived nanovesicles (RDNVs). For instance, a study by Wan et al. (2024) [29] explored the use of RDNVs as drug carriers for macrophage-targeted delivery, demonstrating that these nanovesicles, derived from both wild-type (WT-RDNVs) and CD47 knockout (KO-RDNVs) mouse RBCs, efficiently deliver drugs into macrophages both in vitro and in vivo. This preclinical evidence underscores the therapeutic potential of erythrocyte ghost-based carriers for targeted drug delivery, while also illustrating the need to balance CD47-mediated immune evasion and liver-specific targeting to avoid exacerbating liver damage in conditions like sepsis.

Despite the growing interest in biomimetic nanocarriers, most current strategies remain focused on prolonging systemic circulation and evading immune recognition. Comparatively little attention has been directed toward exploiting disease-specific alterations in physiological clearance pathways to guide carrier biodistribution. In abdominal sepsis, systemic inflammation profoundly alters erythrocyte turnover and reshapes the hepatic immune microenvironment, thereby creating a unique opportunity for disease-guided targeting.

In this review, we propose a conceptual framework in which erythrocyte-derived biomimetic carriers are engineered to harness inflammation-driven hepatic clearance for targeted drug delivery. We examine how sepsis-associated pathophysiological changes influence carrier distribution; discuss major translational challenges, including manufacturing complexity and biological variability; and outline design principles for erythrocyte ghost-based systems tailored to sepsis-induced liver failure. Under certain pathological conditions, physiological clearance may serve not as a barrier to drug delivery, but as a mechanism for selective therapeutic targeting.

Accordingly, we critically evaluate current liver-targeted delivery strategies for sepsis-induced liver failure while highlighting the largely underexplored potential of erythrocyte ghost-based systems. By reframing hepatic clearance not merely as a pharmacokinetic obstacle but as a potential therapeutic gateway, we propose a disease-oriented approach to nanomedicine design for septic organ failure.

## 2. Pathophysiology of Liver Failure in Abdominal Sepsis

Liver failure in sepsis arises from a tightly interconnected cascade of immune dysregulation, microcirculatory collapse, and metabolic stress rather than from direct pathogen-mediated cytotoxicity alone [30]. In abdominal sepsis, continuous translocation of microbial products from the peritoneal cavity and gut exposes the liver to high concentrations of pathogen-associated molecular patterns (PAMPs), including lipopolysaccharide, as well as damage-associated molecular patterns (DAMPs) released from injured host tissues [31,32]. Owing to its anatomical position and dual blood supply, the liver becomes an early and sustained target of these inflammatory signals.

Innate immune sensing represents the initiating step in septic liver injury. Hepatocytes, Kupffer cells, and liver sinusoidal endothelial cells (LSECs) express pattern-recognition receptors such as toll-like receptors (TLRs) on the cell surface and nucleotide-binding oligomerization domain (NOD)-like receptors (NLRs) within the cytosol [33,34]. Engagement of these receptors activates downstream signaling pathways, most prominently NF-κB and inflammasome complexes, leading to robust production of proinflammatory mediators including tumor necrosis factor-α (TNF-α), interleukin-1β (IL-1β), and interleukin-6 (IL-6) [35,36,37]. While initially protective, sustained activation of these pathways amplifies local inflammation and disrupts hepatic immune homeostasis.

The inflammatory response in sepsis is characterized by the coexistence of pro- and anti-inflammatory signals rather than a linear progression [38]. Early in disease, a type 1 immune profile predominates, marked by the recruitment of neutrophils, inflammatory monocytes, and classically activated (M1) macrophages. These cells produce interferon-γ, TNF-α, reactive oxygen species, and chemokines that exacerbate hepatocellular injury and sinusoidal dysfunction [39]. As sepsis evolves, the immune system attempts to restore balance by shifting toward a type 2 response dominated by alternatively activated (M2) macrophages, innate lymphoid cells, and natural killer T cells. Cytokines such as IL-4, IL-5, and IL-13 promote tissue repair and resolution of inflammation; however, when dysregulated, this reparative program contributes to fibrogenesis and the persistent impairment of liver function [33,35].

Concurrently, inflammatory signaling alters the hepatic microenvironment. Endothelial activation and nitric oxide overproduction disrupt the sinusoidal architecture, leading to impaired oxygen delivery and mitochondrial dysfunction in hepatocytes [40,41]. Capillary leak syndrome and hypoalbuminemia further compromise intravascular oncotic pressure, while the altered expression of hepatic transporters and metabolic enzymes distorts drug handling [42,43]. Activated Kupffer cells and LSECs exhibit heightened phagocytic activity, accelerating the clearance of circulating particulates and drug carriers [44,45].

**Figure 1:**
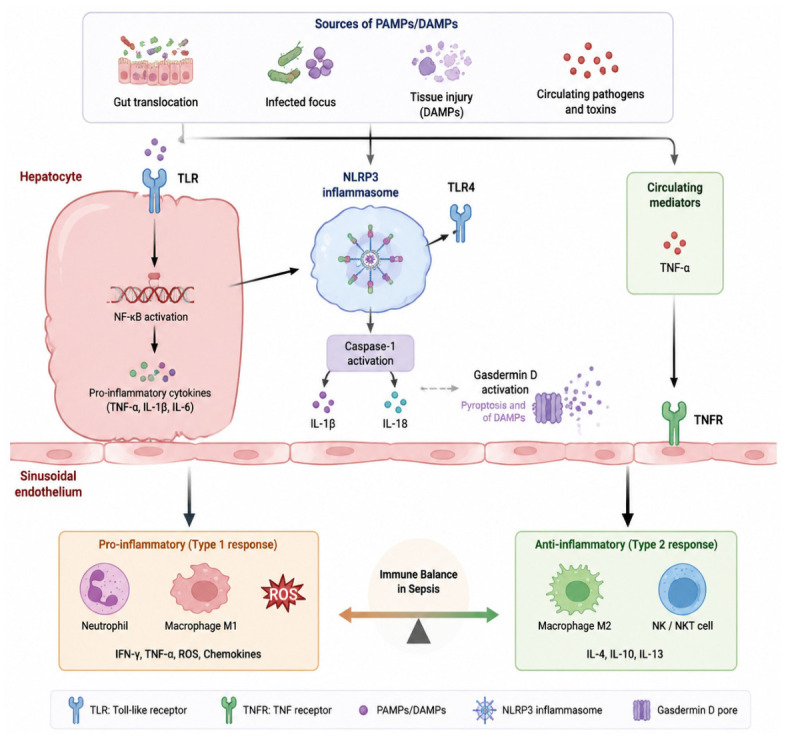
Pathophysiology of liver failure in abdominal sepsis. PAMPs and DAMPs from infection and tissue injury activate TLR/NF-κB and NLRP3 inflammasome pathways in hepatocytes and Kupffer cells, causing pyroptosis, cytokine release, oxidative stress, and immune imbalance, which together drive sinusoidal endothelial injury, hepatocellular dysfunction, and impaired hepatic clearance (created in BioRender. Inuwa, I. (2026) https://BioRender.com/5i2sske, accessed on 7 May 2026). Abbreviations: ROS, reactive oxygen species; TNF-α, tumor necrosis factor-alpha; IL-1β, interleukin-1 beta; PAMPs, pathogen-associated molecular patterns; DAMPs, damage-associated molecular patterns; TLRs, trigger toll-like receptors; NLRs, nucleotide-binding oligomerization domain-like receptors; NF-κB, nuclear factor kappa-light-chain-enhancer of activated B cells; NLRP3, nucleotide-binding oligomerization domain-like receptor protein 3; LPS, lipopolysaccharides.

Collectively, these processes culminate in a hostile and highly dynamic hepatic milieu in which inflammation, immune adaptation, and microvascular failure coexist. Importantly, the same mechanisms that drive septic liver injury also undermine conventional therapeutic strategies [46,47]. Increased vascular permeability, unpredictable receptor expression, and enhanced macrophage-mediated clearance severely limit the effectiveness of systemically administered drugs and synthetic nanocarriers [48]. Understanding this pathophysiological framework is therefore essential for the rational design of liver-targeted delivery systems capable of functioning under septic conditions. A schematic representation of these interconnected mechanisms is presented in Figure 1.

## 3. Challenges in Liver-Targeted Drug Delivery

The development of liver-targeted drug delivery systems remains challenging due to the heterogeneous expression of cellular receptors and complex signaling pathways associated with liver diseases. Beyond serving as a central metabolic organ, the liver functions as a major immunological filter, rapidly removing foreign materials from the circulation. These physiological functions, while protective, severely limit the effectiveness of systemically administered therapeutics [49,50,51]. In particular, rapid drug clearance, strong biological defense mechanisms, and limitations in current delivery technologies further complicate precise hepatic targeting. As a result, current research focuses on understanding these barriers and combining multiple complementary strategies rather than relying on a single solution.

The liver is composed of hepatocytes, Kupffer cells, hepatic stellate cells, and LSECs [52]. Kupffer cells and LSECs are key components of the reticuloendothelial system. They rapidly recognize and clear nanoparticles and drug carriers from circulation before the payload reaches its intended target [53]. In a study by Tsoi et al. (2016) [51], the clearance mechanisms of hard nanomaterials in the liver were examined. The study demonstrated that multiple hepatic cell types, including Kupffer cells, endothelial cells, and B cells, contribute to the uptake and sequestration of these nanomaterials in the liver. This finding challenges the previous assumption that only Kupffer cells are primarily responsible for nanomaterial clearance. Instead, the study revealed that liver endothelial cells and B cells also play a significant role in nanomaterial sequestration, further complicating liver-targeted drug delivery [51]. Drug delivery is further hindered by cytochrome P450 enzymes, which can rapidly metabolize active compounds. This premature metabolism may reduce therapeutic efficacy and, in some cases, cause off-target toxicity [54]. In addition, receptor expression varies between liver cell types and can change during disease progression. This variability complicates the development of a universal targeting system.

Carrier design introduces additional challenges. Particle size, surface charge, and hydrophobicity must be carefully optimized. Large or highly adhesive particles are rapidly cleared, while very small particles often lack selective cellular uptake. Another major limitation is the formation of a protein corona. Blood proteins adsorb onto the nanoparticle surface and promote macrophage recognition and clearance, reducing drug availability at the target site [55]. For instance, a study by Cai et al. (2016) [56] demonstrated that gold nanorods (AuNRs), when pre-incubated with mouse serum, formed a protein corona, which enhanced their liver-targeting ability by preventing premature clearance by Kupffer cells. The study showed that the protein corona, particularly the presence of serum albumin, provided a “stealth” effect, enabling the nanoparticles to escape macrophage recognition, increasing their retention in the liver [56]. This finding underscores the critical role of protein corona formation in modulating nanoparticle behavior and emphasizes its importance in improving liver-targeted drug delivery systems. Moreover, overlapping receptor expression among liver cell populations can result in nonspecific uptake, lowering therapeutic efficiency and increasing the risk of adverse effects [57].

Liver-targeted therapy is hindered by all of the above-mentioned factors; however, it is unlikely that a single strategy will solve all of them, and it seems likely that combining multifunctional nanocarriers with receptor-specific ligands, biomimetic disguises, and computational tools is the most practical next step.

### Translational Implications of the Delivery Barrier in Septic Liver Failure

Understanding the hepatic delivery barrier in sepsis carries direct implications for how delivery systems should be conceptualized, tested, and advanced toward clinical use. A recurring pattern in the nanomedicine literature is the disconnect between preclinical promise and clinical utility—a gap that is especially pronounced in acute inflammatory conditions. The majority of liver-targeted nanocarrier studies have been conducted in tumor-bearing or chemically injured animal models under physiologically stable conditions, where vascular integrity is preserved, receptor expression is predictable, and macrophage activation is confined rather than systemic [49,50]. These conditions bear little resemblance to the hepatic milieu of a septic patient in an intensive care unit, where capillary leak, complement hyperactivation, altered plasma protein composition, and dynamic immune reprogramming fundamentally reshape carrier–tissue interactions [58,59].

From a translational standpoint, this mismatch has three critical consequences. First, efficacy data generated in stable-disease models are likely to overestimate hepatic drug accumulation and underestimate off-target clearance in septic conditions. Carriers that demonstrate sustained hepatic retention in healthy or tumor-bearing mice may be rapidly sequestered by the spleen or cleared by activated circulating monocytes in a septic host, dramatically altering biodistribution profiles [60,61]. Second, receptor-mediated targeting strategies validated in quiescent hepatocytes—such as ASGPR-directed galactosylated carriers—face a fundamentally altered receptor landscape during sepsis, where inflammatory signaling can downregulate ASGPR expression and redirect endocytic machinery toward innate immune responses [62,63]. Targeting assumptions derived from healthy biology therefore require explicit re-evaluation under inflammatory conditions before translational relevance can be claimed. Third, the protein corona that forms on nanocarrier surfaces in septic plasma differs substantially in composition from that in healthy serum, with elevated complement proteins, fibrinogen, pentraxins, and acute-phase reactants that collectively accelerate opsonization and hepatic sequestration through pathways distinct from those observed under normal physiological conditions [64,65].

These translational realities argue strongly for a paradigm shift in how liver-targeted delivery systems are designed and evaluated for inflammatory indications. Characterization studies performed exclusively in healthy biological fluids or stable disease models should be supplemented—ideally replaced at the preclinical stage—by evaluation in septic plasma, lipopolysaccharide-stimulated macrophage co-cultures, and validated murine models of polymicrobial sepsis. Only by grounding carrier design in the biology of the target disease state can translational predictions be made with meaningful confidence. This principle underpins the design framework developed in subsequent sections of this review.

## 4. Liver-Targeted Drug Delivery Strategies

Experimental treatments for liver diseases, including liver failure in abdominal sepsis, are often administered through systemic routes. However, these approaches often result in suboptimal pharmacokinetics and undesired off-target effects [66]. One early strategy developed to overcome these limitations was the use of pharmacocytes—a term applied to describe drug-loaded erythrocyte ghosts or erythrocyte-based cellular carriers for targeted therapeutic delivery [67]. In this context, pharmacocytes can be defined as cell-derived carriers that use cellular or cell-like structures to transport therapeutic agents with improved specificity to target sites. This platform extends the circulation time of drugs, mitigates off-target activity, and minimizes the risk of immune reactions [68,69].

Contemporary strategies focus on refining the precision of drug delivery techniques, addressing the challenges of earlier, more generalized methods. The primary challenge is not the concept but rather ensuring adequate drug reach the damaged liver tissue without compromising other organs [66]. Free circulating drugs in the blood lose a large percentage of the drug before reaching the target site, limiting therapeutic efficiency and increasing side effects, especially in severe cases, such as liver failure in abdominal sepsis, where traditional delivery methods are severely limited [68].

**Figure 2:**
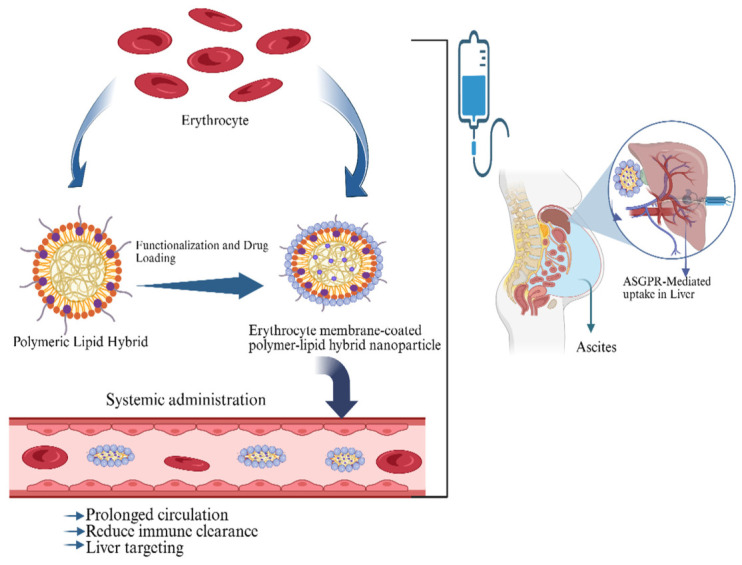
Liver-targeted drug delivery via ASGPR-mediated uptake. Ligand-modified nanoparticle selectively bind to ASGPR expressed on hepatocyte membranes, facilitating endocytosis and intracellular drug release. This strategy enhances hepatic drug accumulation while reducing systemic toxicity and improving therapeutic efficiency (created in BioRender. Inuwa, I. (2026) https://BioRender.com/xlzdyzp, accessed on 7 May 2026). Abbreviations: ASGPR, asialoglycoprotein receptor.

Given the liver’s unique anatomical features, including its blood supply, specialized micro-vessels (sinusoids), and selective entry points such as the asialoglycoprotein receptor (ASGPR), advancements in nanotechnology leverage these natural advantages for targeted drug delivery and facilitating drug access to the liver [70]. Liposomes and polymeric nanoparticles are widely used to protect drugs from enzymatic degradation and enable controlled release. However, issues such as immunogenicity, complement activation, and poor targeting specificity persist. Recent advancements in biomimetic drug delivery have shown promise in overcoming these limitations. For example, polymer–lipid hybrid nanoparticles can be engineered to selectively engage ASGPR on hepatocytes, thereby enhancing targeting precision (Figure 2). Active targeting approaches further improve precision through surface modification with ligands such as antibodies, peptides, or small molecules. These ligands bind specific receptors, including the ASGPR on hepatocytes [69]. Additionally, attention is also directed toward other receptors expressed on Kupffer cells and LSECs, such as Fc receptors and organic ion transporters, particularly in inflammatory and fibrotic liver conditions [52,54]. Alternative routes of administration are also being explored. Oral delivery systems that exploit lymphatic transport or receptor-mediated transcytosis may reduce the need for injections [71]. In parallel, computational modeling and AI-based simulations are emerging as valuable tools for predicting carrier–biological interactions and optimizing design before experimental validation [66].

### 4.1. Conventional Nanocarriers

Nanotechnology-based drug delivery systems, including liposomes, polymeric nanoparticles, and micellar formulations, have been extensively explored for liver targeting. These carriers can improve solubility, protect drugs from premature degradation, and enable controlled release. In models of chronic liver disease and oncology, such systems have demonstrated enhanced hepatic accumulation through passive mechanisms such as the enhanced permeability and retention effect or through receptor-mediated uptake [72].

However, their performance in sepsis-induced liver failure has been inconsistent. Systemic inflammation promotes rapid opsonization and protein corona formation, marking nanoparticles for clearance by activated macrophages [58,59]. In addition, receptor expression patterns on hepatocytes and non-parenchymal cells are often disrupted during sepsis, undermining the reliability of ligand-based targeting strategies [61]. As a result, many synthetic carriers fail to achieve sustained hepatic drug levels precisely when they are most needed.

Micellar formulations, despite their well-documented stealth properties and extended systemic circulation in stable disease models, face compounding limitations in the septic environment. Structurally, conventional micelles exist only above a critical micellar concentration (CMC) and disassemble when diluted below this threshold upon systemic administration; native plasma proteins such as albumin further destabilize micellar integrity at physiological concentrations, promoting premature drug release before hepatic accumulation can occur [16,73]. In sepsis, this structural vulnerability is compounded by a dramatically shifted plasma proteome—elevated complement proteins, fibrinogen, and acute-phase reactants—that rapidly remodels micellar surface chemistry into an opsonization-prone corona, overriding PEG-based stealth coatings regardless of their engineering. For instance, Shaw et al. (2025) in their study, demonstrated that inflammatory disease progression alters nanoparticle corona composition in a disease-dependent manner, directly modifying innate immune cell interactions and cytokine responses beyond what healthy-condition stealth coatings can counteract [74]. Critically, micelles rely on passive accumulation mechanisms, primarily the enhanced permeability and retention (EPR) effect that are mechanistically unavailable in the generalized vascular dysfunction of sepsis, as discussed in Section 4.3. Perhaps most fundamentally, the design logic of conventional micelles is misaligned with the biology of septic liver failure: they are engineered to evade macrophage recognition, whereas the therapeutically relevant target in this setting is the activated Kupffer cell. A delivery system that bypasses this effector cannot modulate the intracellular NF-kB signaling, cytokine amplification, and inflammasome activity that drive septic hepatic injury at their source.

To take advantage of both materials, hybrid delivery systems have been developed that combine the properties of lipids and polymers, providing for greater stability over time, increased drug encapsulation, and improved control over drug release profiles [75]. These characteristics are particularly important in conditions like sepsis, in which timing and dosage concentration play a significant role in therapeutic outcomes. Additionally, some hybrid carriers can be designed to release drugs only in specific pathological conditions, since some carriers are responsive to the environment, such as pH changes [70].

### 4.2. Active Targeting Approaches

To overcome nonspecific distribution, active targeting strategies have focused on exploiting liver-specific receptors. To enable drug carriers to selectively bind to the ASGPR on liver cells, a targeted tactic is to attach them with galactose molecules [76]. In damaged liver tissues, certain carriers passively accumulate due to the enhanced permeability and retention (EPR) effect; however, others carriers are made to be actively targeted by recognizing liver receptors. Other approaches target scavenger receptors, Fc receptors, or organic ion transporters expressed by Kupffer cells and liver sinusoidal endothelial cells. For instance, in mouse cancer models, nanoparticles designed to deliver simvastatin preferentially enter LSECs, reverse endothelial capillarization, and remodel the tumor microenvironment, enhancing anti-tumor immunity and slowing hepatocellular carcinoma progression [77]. Another recent preclinical platform used peptide-conjugated nanoparticles that home to LSECs to induce antigen-specific immune tolerance in multiple autoimmune disease models, demonstrating the potential of LSEC-targeted delivery for precision immunotherapy [78].

On the Kupffer cell side, lipidoid nanoparticles carrying siRNA against PD-L1 were shown in mice to be predominantly taken up by Kupffer cells, silencing PD-L1 expression and boosting local NK and CD8^+^ T cell responses during viral infection, underscoring how Kupffer cell-specific uptake can be harnessed for targeted immune modulation [79].

While conceptually attractive, these strategies rely on relatively stable receptor expression and preserved endocytic function; conditions that are frequently compromised during sepsis. Inflammatory signaling, hypoxia, and metabolic stress can downregulate receptor availability or redirect carrier uptake toward non-therapeutic pathways. Consequently, receptor-based targeting alone has not resolved the fundamental delivery challenges posed by septic liver injury.

### 4.3. Translational Assessment of Current Targeting Strategies in Sepsis

A rigorous translational appraisal of existing liver-targeted delivery strategies reveals a consistent pattern: systems designed for precision receptor engagement under stable physiological conditions exhibit diminished targeting fidelity and unpredictable biodistribution when deployed in septic environments. This section moves beyond reporting study outcomes to critically evaluate the underlying technological concepts and their intrinsic translational limitations in the context of abdominal sepsis.

Passive targeting through the EPR effect, which has underpinned hepatic nanocarrier accumulation strategies in oncology, is not reliably operative in sepsis-induced liver failure. It is worth noting that the EPR effect is itself size-dependent. Nanoparticles in the 10–200 nm range are generally considered optimal for passive accumulation through tumor vascular fenestrations, while particles exceeding 200 nm are predominantly sequestered by the mononuclear phagocyte system in the liver and spleen, and those below approximately 8 nm are subject to rapid renal filtration [80]. The EPR effect depends on sustained local vascular hyperpermeability combined with impaired lymphatic drainage—conditions that are tumor-specific and not reproducible in the generalized vascular dysfunction of systemic sepsis [72]. In the septic liver, increased vascular permeability is accompanied by endothelial glycocalyx degradation, sinusoidal architectural disruption, and dynamic fluctuations in microvascular flow that collectively produce unpredictable rather than enhanced drug accumulation [81,82]. Therefore, designing carriers for passive hepatic retention based on EPR principles carries a fundamental translational risk when the target indication is septic organ failure rather than solid tumor disease.

Active targeting through ASGPR-directed ligands—the most clinically explored liver-specific targeting mechanism—faces a comparable conceptual limitation. ASGPR-mediated endocytosis is a hepatocyte-restricted, clathrin-dependent process that requires both adequate receptor surface density and functional endocytic machinery [83]. Both of these prerequisites are compromised during sepsis: ASGPR expression is suppressed by inflammatory cytokines including TNF-α and IL-1β through NF-κB-mediated transcriptional repression [62], while hepatocyte endocytic capacity is reduced by mitochondrial dysfunction and metabolic stress [5,46]. A galactosylated carrier that efficiently homes to hepatocytes in a fibrotic or tumor-bearing liver may therefore exhibit markedly reduced uptake in a septic liver where its target receptor is functionally impaired. Critically, this limitation is rarely acknowledged in preclinical publications that use ASGPR-targeting under non-inflammatory conditions and then extrapolate findings to inflammatory liver disease.

Kupffer cell-targeted strategies present a more nuanced translational picture. Unlike ASGPR-based approaches, targeting strategies that leverage scavenger receptors, complement receptors, and Fc receptors on Kupffer cells may actually benefit from the heightened macrophage activation that characterizes sepsis [79]. Kupffer cells in the septic liver exhibit upregulated pattern recognition receptor expression, enhanced phagocytic activity, and amplified complement receptor engagement, creating a permissive environment for particulate carrier uptake [84,85]. The translational challenge here is not receptor availability but rather therapeutic consequence: delivery of drug cargo into hyperactivated Kupffer cells must be designed to modulate rather than further amplify their inflammatory output. Agents such as anti-inflammatory lipid mediators, NF-κB inhibitors, siRNA targeting TNF-α or IL-1β, or mitochondria-targeted antioxidants represent therapeutically rational payloads for this route, provided that intracellular release kinetics are calibrated to the timeline of macrophage activation in sepsis [59,86,87].

From a manufacturing and clinical feasibility perspective, conventional nanocarriers—including liposomes, polymeric nanoparticles, and lipid nanoparticles—currently offer the most scalable and regulatorily tractable platform for liver-targeted sepsis therapy. Several liposomal and lipid nanoparticle formulations have been approved for systemic administration, providing a regulatory template that can be adapted for hepatic targeting [58,88]. However, translating these systems to acute sepsis requires addressing formulation stability under the altered pH and ionic conditions of septic plasma, compatibility with concomitant medications common in intensive care (including vasopressors, broad-spectrum antibiotics, and sedatives), and the feasibility of intravenous administration in hemodynamically unstable patients. These operational considerations are rarely addressed in preclinical nanomedicine studies but are decisive in determining whether a delivery system can progress from bench to bedside in an acute critical illness setting.

In aggregate, this translational analysis identifies a hierarchy of strategies: ASGPR-targeted approaches are most reliable in stable hepatocellular disease but least dependable in acute septic injury; passive EPR-based accumulation is mechanistically inappropriate for sepsis; and Kupffer cell-exploiting strategies, despite their conceptual alignment with septic biology, require careful payload selection and release engineering to yield therapeutic rather than harmful outcomes. This hierarchy informs the biomimetic framework developed in the subsequent sections.

## 5. Core Design Principles of Biomimetic Targeted Drug Delivery

Biomimetic drug delivery systems are designed by emulating the structural, functional, and biological features of natural systems to overcome the intrinsic limitations of conventional synthetic nanoparticles [89]. Unlike traditional nanoparticles, which often face rapid immune clearance, limited targeting efficiency, and poor biological integration, biomimetic platforms leverage nature-derived components to enhance circulation stability, targeting specificity, and therapeutic efficacy [90,91]. The key design principles are outlined below.

I. Biological Camouflage and Immune Evasion

Herein, cell membrane-coated nanoparticles, derived from erythrocytes, platelets, leukocytes, or cancer cells, inherit surface proteins and antigens from their source cells, reducing opsonization and uptake by the reticuloendothelial system [92,93]. This represents a significant advantage over PEGylated systems, which may still trigger immune recognition or accelerated blood clearance upon repeated administration [93]. Biomimetic carriers achieve immune evasion through intrinsic biological identity rather than artificial stealth coatings [94].

II. Biologically Driven Active Targeting

This is another fundamental biological principle that works through natural recognition mechanisms. Biomimetic systems utilize endogenous ligand–receptor interactions to achieve active targeting without extensive synthetic functionalization [95]. For instance, platelet membrane-coated nanoparticles can selectively adhere to damaged vasculature or inflamed tissues, while cancer cell membrane-derived carriers exhibit homotypic targeting toward tumors of the same origin [95,96,97,98]. This biologically driven targeting strategy contrasts with conventional nanoparticles that rely heavily on synthetic ligands, antibodies, or peptides, which often suffer from instability, limited binding efficiency, and batch-to-batch variability. Biomimetic targeting therefore offers a more robust and physiologically relevant approach to site-specific drug delivery [22,99].

III. Structural and Mechanical Mimicry

Natural biological entities possess optimized sizes, shapes, and mechanical properties that facilitate circulation, tissue penetration, and cellular uptake [100,101]. By mimicking these features, biomimetic nanoparticles can better navigate biological barriers such as vascular endothelium, mucus layers, and cellular membranes. Additionally, functional mimicry, such as enzyme-responsive behavior or pH-sensitive drug release, allows biomimetic systems to respond dynamically to disease-specific microenvironments [86,87,96,102]. These adaptive characteristics are difficult to achieve with single-component synthetic nanoparticles, highlighting the superiority of biomimetic designs in complex pathological settings.

IV. Rational Material Hybridization

Material selection is another critical design consideration, as biomimetic drug delivery systems often integrate natural, recombinant, or bio-derived components with synthetic cores to balance biological functionality and physicochemical stability [100,103]. While natural membranes and biomolecules provide biocompatibility and targeting capability, synthetic materials contribute structural integrity, controlled drug loading, and scalable manufacturing. The rational hybridization of these elements enables the development of multifunctional platforms that outperform purely synthetic nanoparticles in both in vivo performance and translational potential [104].

V. Physiological Compatibility and Reduced Immunogenicity

Unlike conventional solid particles that may trigger foreign body responses or liposomal formulations that can activate complement pathways, biomimetic carriers present endogenous surface signals that promote physiological compatibility [105]. This principle is particularly critical in inflammatory conditions such as sepsis, where immune hyperactivation amplifies the immunogenic potential of synthetic carriers.

Overall, the design principles of biomimetic drug delivery emphasize harmony with biological systems rather than the forceful manipulation of them. By incorporating immune evasion, natural targeting, structural optimization, and responsive functionality into a single platform, biomimetic carriers may address key limitations of traditional nanoparticles. These principles not only enhance therapeutic outcomes but also provide a conceptual framework for the next generation of drug delivery systems aimed at clinical translation [105].

## 6. Biomimetic Platforms: Types and Rational Design

**Figure 3:**
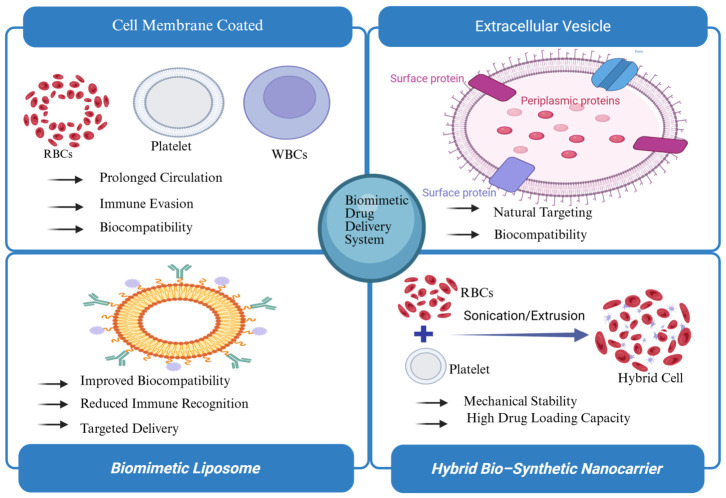
Types of biomimetic drug delivery systems. Overview of major biomimetic nanocarrier platforms developed to enhance drug delivery performance. These systems mimic natural biological structures, including cell membrane-coated nanoparticles, extracellular vesicle carriers, biomimetic liposome, and hybrid platform, enabling prolonged circulation time, immune evasion, and targeted delivery to diseased tissues such as the liver (created in BioRender. Inuwa, I. (2026) https://BioRender.com/1pl8z19, accessed on 22 May 2026). Abbreviations: RBCs, red blood cells; WBCs, white blood cells.

Based on the fundamental design principles outlined above, several biomimetic platforms have been developed to address specific delivery challenges that conventional nanoparticles fail to overcome. These platforms integrate biological functionality with rational material engineering, resulting in hybrid systems that exhibit enhanced circulation, targeting, and therapeutic performance. A schematic representation of the major biomimetic platforms discussed in this section is presented in Figure 3.

### 6.1. Cell Membrane-Coated Nanoparticles

Cell membrane-coated nanoparticles represent one of the most studied biomimetic platforms. In this approach, synthetic nanoparticle cores, typically composed of polymers, lipids, or inorganic materials, are cloaked with natural cell membranes derived from erythrocytes, platelets, leukocytes, or cancer cells [20,21,106]. The resulting core–shell structure retains the physicochemical advantages of synthetic nanoparticles while inheriting the biological identity of the source cells [107,108].

Erythrocyte membrane-coated nanoparticles (RBC-coated NPs) are created by coating synthetic nanoparticle cores, such as polymers, lipids, or metals, with natural red blood cell membranes [109]. These membranes are processed into nanoscale vesicles using techniques like sonication, extrusion through porous membranes, or electroporation, breaking them into tiny, flexible fragments [110,111]. These fragments are then fused with the synthetic nanoparticle cores. A common method for this fusion is co-extrusion, where the membrane and nanoparticle mixture are forced repeatedly through a porous membrane, wrapping the erythrocyte membrane around the core [112,113]. RBC-coated nanoparticles are distinguished by prolonged systemic circulation and immune evasion, attributed to surface self-recognition markers, such as CD47, that inhibit macrophage-mediated phagocytosis [110,114]. Li et al. demonstrated that bFGF-loaded erythrocyte membrane-coated nanoparticles attenuated sepsis-induced cardiac injury by preserving cardiomyocyte viability, highlighting their therapeutic potential beyond hepatic applications [115].

As a critical player in hemostasis, wound healing, inflammation, and other biological functions, platelets are highly responsive cells that adapt to environmental changes [116,117]. They release a variety of soluble biomolecules, including growth factors, coagulant factors, and extracellular vesicles, which are essential for tissue repair and immune modulation [95,108,118]. Additionally, platelets possess the unique ability to evade immune detection, adhere to sub-endothelial surfaces, and interact with pathogens [119,120]. These inherent properties have inspired the creation of platelet membrane-coated nanoparticles (PNPs), which harness platelet membranes to enhance drug delivery systems, offering a promising strategy for targeted therapy [120,121,122]. For example, a recent study developed platelet membrane-coated mesoporous silica nanoparticles (MSNs) that combine a vascular disruption agent (VDA) with an anti-angiogenic drug (AAD) for targeted tumor treatment. In this platform, the nanoparticles efficiently accumulate at tumor sites via platelet membrane adhesion to damaged vessels, enhancing vascular disruption and anti-angiogenesis in animal models [123]. However, despite their potential, this approach faces challenges, such as the risk of inducing angiogenesis in the later stages of treatment, which could lead to tumor recurrence. Additionally, the nonspecific targeting of VDAs and potential side effects limit their clinical applicability.

Although nanoparticle drug delivery systems have shown potential in cancer therapy, challenges such as susceptibility to immune clearance and inefficient targeting in complex intercellular environments persist. To overcome these limitations, cancer cell membrane-encapsulated nanoparticles (CCM-NPs) have been developed using biomimetic nanotechnology [124]. By utilizing the proteins found on the surface of cancer cell membranes, CCM-NPs gain several beneficial properties, including immune evasion and homologous cell recognition. Additionally, the surface of the cancer cell membrane is enriched with tumor-specific antigens, enabling the CCM-NPs to deliver these antigens, activate a downstream immune response, and effectively combat tumors [125].

From a design perspective, membrane coating techniques such as extrusion, sonication, or microfluidic assembly must preserve membrane protein orientation and functionality [126]. The rational selection of membrane source, core material, and coating method is therefore critical to achieving reproducible and biologically active systems. A comparison of cell membrane-coated nanoparticle systems for biomimetic drug delivery is presented in Table 1.

### 6.2. Extracellular Vesicle and Exosome-Inspired Systems

Extracellular vesicles (EVs) are membrane-bound vesicles secreted by cells into the extracellular space [144]. They are classified into three main types—micro-vesicles (MVs) exosomes, and apoptotic bodies—which are distinguished by their formation processes, release mechanisms, size, content, and functions [145,146]. EVs are natural nanocarriers that mediate intercellular communication by transporting proteins, lipids, and nucleic acids. Their inherent biocompatibility, low immunogenicity, and intrinsic targeting ability make them attractive candidates for drug delivery [147,148]. However, challenges related to low yield, heterogeneity, and scalability have limited their widespread clinical translation.

To address these issues, exosome-inspired biomimetic systems have been developed, combining synthetic vesicles with selected EV-like features. These hybrid platforms aim to replicate the size, lipid composition, and surface functionality of natural exosomes while enabling scalable production and controlled cargo loading [91,149].

### 6.3. Biomimetic Liposomes

Liposomes remain a clinically validated drug delivery system, and recent advances have transformed them into biomimetic platforms through the incorporation of biological components [104]. Liposomes are bilayer structures with physiological compatibility similar to that of human cell membranes, making them compatible with cell membranes. Depending on their lipid compositions and preparation methods, the diameter of liposomes ranges from about 20 nm to more than 1 μm [150].

Biomimetic liposomes are lipid-based nanoparticles designed to mimic natural biological membranes. Formed by the self-assembly of lipids into spherical vesicles, these liposomes can be modified with biological components, such as cell membrane proteins or lipids, to enhance their interaction with specific tissues [100,104]. This modification improves their biocompatibility, reduces immune recognition, and allows for targeted drug delivery [151].

One of the key advantages of biomimetic liposomes is their ability to enhance drug targeting and extend circulation time, making them ideal carriers for therapeutic agents such as small molecules, proteins, and nucleic acids [152,153,154]. Their similarity to natural cells helps evade the immune system, potentially reducing side effects and improving therapeutic efficacy [104]. In a recent study, liposomes were modified with glioma cell membrane fragments and successfully crossed the blood–brain barrier (BBB). These drug-loaded biomimetic liposomes showed enhanced accumulation in tumor tissue and improved efficacy of paclitaxel and carboplatin, demonstrating their potential in targeted cancer therapy [155].

However, challenges remain, including high production costs, stability issues, and limited cargo capacity. Biomimetic liposomes may also face immune clearance, especially upon repeated administration, due to complement activation [155,156,157]. Despite these limitations, ongoing research focuses on optimizing their design to overcome these hurdles and expand their use in various medical applications [124,158].

### 6.4. Hybrid Bio-Synthetic Nanocarriers

Recently, the focus on hybrid bio-synthetic nanocarriers has grown, involving the coating of nanoparticles with hybrid membranes from different cell types. The resulting hybrid cell membrane-coated nanoparticles outperform nanoparticles coated with a single type of membrane in terms of mechanical stability, drug loading capacity, and release kinetics, while maintaining biological compatibility [159]. Although cell membrane coating significantly improves the functionality of nanoparticles, additional modifications are often required to optimize their performance for targeted applications. While cell membrane coatings provide immune evasion, adding targeting ligands can further enhance the precision of delivery, enabling NPs to selectively target specific sites, such as tumors [93,160].

To improve the functionality of CNPs, one approach involves combining membranes from different cell types and applying them to the nanoparticle surface. By fusing living cells to create hybrid cells, their membranes can be harvested and used to coat the nanoparticles. This process can be facilitated through methods like electrofusion or polymer-induced aggregation, which have been extensively studied to promote cell fusion [161,162]. Alternatively, sonication or extrusion can be used to merge cell membranes from distinct sources, which are then applied to the NP substrates. Both techniques ensure that the specific properties of each cell type are preserved on the nanoparticles, enabling enhanced functionality [163]. For instance, Dehaini et al. (2017) [163] demonstrated how red blood cell and platelet membranes were fused to coat nanoparticles, creating a dual-membrane platform that combines immune evasion from erythrocytes with vascular targeting from platelets. The resulting hybrid nanoparticles retained key characteristics from both cells, offering prolonged circulation and enhanced biocompatibility, showcasing how membrane fusion can enhance nanoparticle functionality beyond single-membrane systems [163].

In the case of liver failure associated with abdominal sepsis, this technology is particularly critical. Targeted delivery is essential to ensure that therapeutic agents reach the liver tissue while minimizing the side effects. By coating nanoparticles with biologically relevant membranes, hybrid bio-synthetic nanocarriers can facilitate precise targeting of damaged liver tissue, allowing for the direct delivery of therapeutics. This precision is especially important in abdominal sepsis, where the liver is an early and sustained target of inflammatory signals. Such targeted strategies can improve therapeutic efficacy in liver repair and immune modulation, offering significant advantages over conventional treatments in the context of sepsis-induced liver dysfunction.

## 7. Autologous Erythrocyte Ghosts as Liver-Targeted Drug Delivery Platforms

### 7.1. Structure and Functions of Red Blood Cells

Erythrocytes have long served as a model system for membrane biology and have become increasingly important in drug delivery because of their abundance, biocompatibility, deformability, long circulation time, and lack of nuclei or intracellular organelles [164,165,166,167]. Their membrane contains a complex lipid–protein architecture that supports mechanical stability, immune recognition, and interaction with macrophage-mediated clearance pathways [168,169]. These properties make erythrocytes particularly attractive for carrier engineering, especially when autologous cells are used to minimize immunogenicity.

For drug delivery, erythrocyte-derived platforms offer two related advantages. First, intact erythrocytes or erythrocyte ghosts can encapsulate therapeutic cargo within a biologically compatible membrane [127,170]. Second, their natural interactions with the mononuclear phagocyte system can be used to guide delivery to clearance organs such as the liver and spleen [169,171,172]. In sepsis-associated liver failure, this feature is especially relevant because hepatic macrophage activation and altered erythrocyte turnover may redirect erythrocyte-derived carriers toward inflamed hepatic tissue. Thus, rather than treating erythrocyte clearance as a limitation, autologous erythrocyte ghost platforms may be designed to exploit this pathway for targeted delivery.

### 7.2. The Role of Erythrocyte Ghosts in Liver-Targeted Drug Delivery

Traditional treatment for liver failure in abdominal sepsis often fails to effectively treat both the infectious and hepatic injury components, leading to high morbidity and mortality rates. Fueled by the need to address this gap in treatment efficacy, scientists have investigated liver-targeted drug delivery, such as nanoparticles, liposomes, and biological carriers. Among these, biological carriers have gained attention due to their inherent biocompatibility and ability to evade immune surveillance.

Recently, using autologous erythrocyte ghosts as one of the biological carriers in targeted drug delivery has become the most innovative delivery method. These emptied red blood cells naturally bind to serum proteins such as apolipoprotein E (ApoE) and are delivered to hepatocytes by binding to low-density lipoprotein (LDL) receptors [173]. The complex microenvironment of the liver, composed of different specialized cell types, also plays a role in the absorption, elimination, and function of this organ [174].

**Figure 4:**
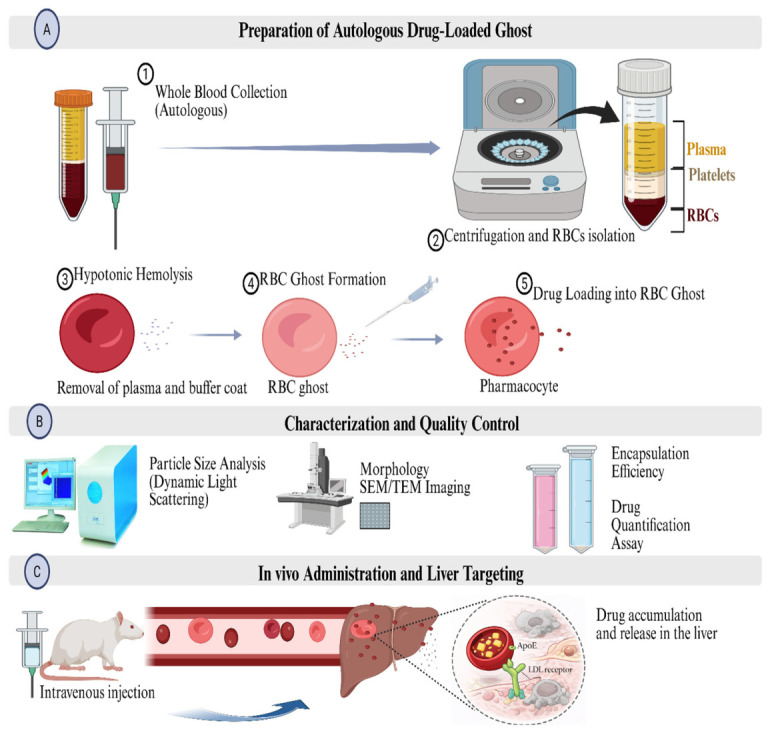
Schematic illustration of drug encapsulation and liver targeting using an autologous erythrocyte ghost. (**A**) Preparation workflow showing blood collection, centrifugation-based separation of blood components, isolation of red blood cells, generation of erythrocyte ghosts, drug loading, and formation of drug-loaded erythrocyte ghosts; (**B**) characterization and quality-control procedures used to evaluate the prepared erythrocyte ghosts, including particle or size analysis, microscopic/morphological assessment, and stability or encapsulation-related testing; (**C**) in vivo administration and liver targeting, showing injection into an animal model, vascular circulation, delivery to the liver, and hepatic uptake mediated by receptor-associated interactions. (created in BioRender. Inuwa, I. (2026) https://BioRender.com/8ll955g, accessed on 7 May 2026). Abbreviations: RBCs, red blood cells; ApoE, apolipoprotein E; LDL, low-density lipoprotein.

The development of an erythrocyte ghost, as shown in Figure 4, is obtained by removing the internal contents of red blood cells, leaving the hollow membrane structure which retains the original configuration of the membrane. This protects the important proteins and lipids that are necessary for biological compatibility and targeted interactions with the body [175]. The unique disc shape of the red blood cells, approximately 7–8 μm in diameter, also give a favorable surface-to-volume ratio [176,177]. The mechanical stability and biological signaling function are supported by membrane proteins that include glycophorins and band 3. Since erythrocyte ghosts can be derived autologously, they have a reduced risk of inducing immune rejection [169]. Their lack of nuclei and major histocompatibility complex (MHC) molecules results in minimal immune system activation [178]. Furthermore, because these carriers have a circulatory lifespan of more than 100 days, they provide prolonged drug release, which lowers the frequency of dosing.

Presently, a variety of techniques are available to encapsulate drugs into the erythrocyte ghosts, including endocytosis, electroporation, and hypotonic-osmosis hemolysis, that temporarily permeate the membrane to load drugs in or entrap them in the membrane [179]. However, efficiency is highly dependent on the specific process, such as osmotic balance, incubation temperature, and chemical additives [180]. In addition to their use as passive carriers, erythrocyte ghosts have an inherent advantage in the treatment of liver diseases, as their surface glycoproteins tend to promote macrophage uptake, particularly in the liver and spleen [173]. Scientists have tried modifying the surface by adding ligands or changing opsonization to improve targeting specificity [181]. There is also evidence that their therapeutic potential is increasing: erythrocyte ghosts loaded with interleukin-1β or the antibiotic ceftriaxone accumulated more in inflamed or infected tissues, and this correlated with better treatment outcomes [182]. Additionally, a study by Yuan et al. (2020) [181] demonstrated that ghosts effectively delivered 18F-fluorodeoxyglucose (FDG) to the liver, where macrophage-mediated uptake was a major determinant of the drug [181].

Overall, the intrinsic biocompatibility, prolonged circulation profile, structural integrity, and organ-targeting propensity of autologous erythrocyte ghosts underscore their value as adaptable platforms for controlled drug delivery. Taken together, these attributes position them as one of the most promising natural carrier systems in nanomedicine, particularly for liver-targeted therapeutic applications.

### 7.3. Translational Shortcomings of Erythrocyte Ghost-Based Drug Delivery

To bridge the gap between preparation techniques and clinical translation, this subsection summarizes how specific limitations in erythrocyte ghost production influence in vivo drug delivery outcomes. Generally, erythrocyte ghost-based carriers are sensitive to preparation conditions. Hypotonic lysis, membrane resealing, surface protein retention, and payload–membrane interactions each introduce variability that persists into the in vivo setting [183,184]. These variations translate into pharmacokinetic unpredictability, potential off-target exposure, and altered hepatic clearance [185]. In sepsis-associated liver injury, consequences are further compounded. Oxidative membrane damage, systemic inflammation, and narrow therapeutic windows collectively reduce the tolerance for preparation-derived error [186,187]. The principal limitations and their clinical consequences are summarized in Table 2.

## 8. Adaptive Clearance Modulation as a Targeting Strategy in Sepsis

**Figure 5:**
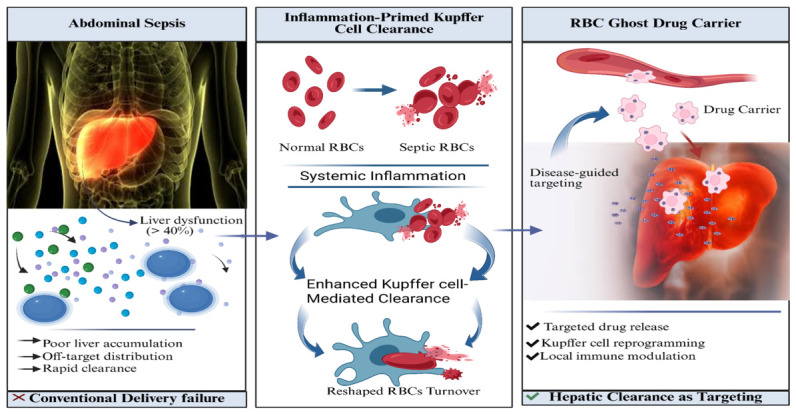
Kupffer cell mediated hepatic clearance as a targeting strategy in sepsis. Schematic illustration showing how inflammation during abdominal sepsis alters circulating red blood cells (RBCs) and enhances their clearance by hepatic Kupffer cells. While conventional drug delivery often fails due to poor liver accumulation and rapid systemic clearance, erythrocyte ghost-based carriers may exploit this pathway to achieve targeted drug delivery and localized therapeutic action in the liver (created in BioRender. Inuwa, I. (2026) https://BioRender.com/ze59myk, accessed on 7 May 2026). Abbreviations: RBCs, red blood cells.

The failure of conventional nanocarriers in septic liver injury underscores a central problem. Most delivery systems are designed for stable physiological environments, whereas sepsis creates an evolving, inflammatory, and clearance-dominated hepatic milieu. In this context, therapeutic success depends less on receptor-specific binding and more on understanding how inflammation reshapes hepatic immune surveillance. We therefore propose an Inflammation-Adaptive Clearance-Guided Targeting (IACGT) framework, summarized in Figure 5, in which carrier design is informed directly by septic liver biology and leverages, rather than resists, disease-amplified clearance mechanisms.

### 8.1. Disease-Driven Design

Rational design must begin with the pathophysiology of septic liver failure. During sepsis, Kupffer cells undergo marked activation characterized by amplified toll-like receptor signaling, NF-κB pathway engagement, inflammasome activation, and excessive cytokine release [84,85]. Single-cell and translational studies confirm that Kupffer cells shift toward inflammatory phenotypes with enhanced phagocytic machinery and complement receptor expression [198,199]. This primed state increases their scavenging efficiency, meaning that circulating particles encounter a liver in a condition for rapid clearance. Systemic inflammation further elevates circulating PAMPs and DAMPs, broadly activating the mononuclear phagocyte system and shortening nanoparticle half-life in vivo [60]. In parallel, septic sinusoidal endothelial cells undergo glycocalyx shedding, adhesion molecule upregulation, and permeability alterations that modify particle margination and trans-sinusoidal transport [81,82].

Under these conditions, classical receptor-driven targeting strategies become unreliable. Receptors such as ASGPR may be downregulated or functionally impaired, while macrophage-mediated clearance becomes dominant, homeostatic signaling may pathways fluctuate [62,63]. Taken together, these alterations establish a clearance-dominant environment in which macrophage sequestration overrides ligand-based targeting precision. Therefore, carrier engineering should therefore prioritize compatibility with inflammatory uptake pathways rather than depend solely on ligand–receptor specificity established under healthy conditions.

### 8.2. Clearance as a Delivery Route

Conventional nanomedicine seeks to evade macrophage uptake to prolong systemic circulation. However, in the context of septic liver failure, this strategy often contradicts the biological reality. RBC-derived systems, in contrast, offer a unique advantage. Erythrophagocytosis, the natural process by which aging or damaged erythrocytes are cleared by the spleen and Kupffer cells, could be harnessed in this setting [200,201]. Under inflammatory conditions, this clearance process is accelerated through oxidative damage to cell membranes, exposure of phosphatidylserine, and changes in erythrocyte deformability [202,203]. Rather than masking these interactions, engineered RBC ghosts can be tuned to preferentially engage activated Kupffer cells. 

During sepsis, complement receptors and scavenger receptors are upregulated on Kupffer cells, providing a biologically accessible internalization route [204]. Once internalized, RBC ghosts may deliver anti-inflammatory agents, antibiotics, nucleic acids, or immunomodulatory compounds directly into macrophage cytosol. This approach shifts the concept of phagocytosis from being a barrier to drug delivery to a targeted entry mechanism, where intracellular signaling pathways—like NF-κB activation or cytokine production—can be modulated at their source. Rather than viewing Kupffer cell uptake as a complication, it could be strategically leveraged as part of the therapeutic design. Ultimately, this strategy reframes hepatic clearance from a pharmacokinetic limitation into a programmable delivery route, as illustrated in Figure 5.

### 8.3. Adaptive Surface Engineering in the Septic Microenvironment

Surface design for liver-targeted delivery in sepsis cannot rely on assumptions derived from healthy immune conditions. Systemic inflammation reshapes both cellular recognition pathways and the plasma protein environment, altering how circulating particles are processed by the liver.

Under physiological conditions, CD47–SIRPα signaling limits macrophage engulfment by transmitting a “self” signal [28,205]. However, during sepsis, inflammatory stress may alter CD47 expression or downstream responsiveness [206], and septic RBCs exhibit oxidative membrane injury, cytoskeletal disruption, and phosphatidylserine externalization [207]. These changes increase susceptibility to macrophage recognition, and this suggests that preservation of CD47 alone is insufficient to guarantee predictable behavior of erythrocyte-derived carriers in inflamed settings.

In parallel, the plasma protein environment shifts dramatically during sepsis, with elevated complement proteins, fibrinogen, and C-reactive protein reshaping nanoparticle protein coronas [64,65]. Complement hyperactivation, which is central to sepsis pathogenesis, drives C3b deposition and enhances hepatic particle uptake [208]. Rather than universally suppressing complement interactions, clearance-guided systems may strategically modulate them to favor macrophage-selective internalization. These changes accelerate hepatic uptake and shift biodistribution patterns. Accordingly, carrier characterization performed exclusively in healthy serum lacks translational relevance; evaluation under septic biological fluids is essential.

Adaptive surface engineering therefore requires a departure from static stealth paradigms. Rather than striving for complete immune evasion, biomimetic carriers should be calibrated for controlled interaction with activated macrophages. This includes the following:

- Consideration of complement-resistant or complement-modulating modifications.
- Evaluation of CD47-mediated signaling in inflamed conditions.
- Fine-tuning membrane composition to balance immune recognition and controlled uptake.

In this framework, excessive immune invisibility may limit intracellular delivery, while uncontrolled opsonization may cause premature systemic clearance. The design objective is not immune escape per se, but controlled biological engagement aligned with inflammatory plasma dynamics. This represents a conceptual shift from stealth engineering toward inflammation-adaptive surface.

### 8.4. Translational Design Roadmap for IACGT-Based Erythrocyte Ghost Carriers

Translating the IACGT framework from conceptual proposition to clinical application requires a structured development pathway that integrates carrier engineering, preclinical validation, and manufacturing science within a framework aligned with regulatory expectations. The following design roadmap outlines the key technological decisions and translational milestones that would govern the development of erythrocyte ghost-based carriers for septic liver failure, drawing directly on the pathophysiological principles outlined in Section 2, Section 8.1, Section 8.2 and Section 8.3.

Stage 1—Disease-Informed Carrier Specification

The first translational step is the definition of carrier target product profiles (TPPs) that are grounded in the biology of septic liver failure rather than derived from stable-disease precedents. Based on the pathophysiology reviewed in Section 2, a clinically relevant erythrocyte ghost carrier for this indication should satisfy the following functional criteria: (i) preferential uptake by activated Kupffer cells rather than circulating monocytes or splenic macrophages; (ii) intracellular payload release timed to the peak inflammatory phase of sepsis, typically within 24–72 h of insult onset [30,201,209]; (iii) surface composition calibrated to complement-enhanced rather than complement-evasive uptake to exploit the complement hyperactivation characteristic of early sepsis [210]; and (iv) mechanical deformability sufficient to navigate sinusoidal constrictions in a liver with altered microvascular architecture [211,212]. These criteria represent a departure from standard erythrocyte ghost design parameters, which have historically been optimized for prolonged systemic circulation rather than targeted inflammatory uptake.

Stage 2—Sepsis-Specific Preclinical Evaluation

Conventional preclinical evaluation of nanocarriers in healthy animals or tumor models is insufficient for establishing translational relevance in acute septic organ failure. IACGT-based erythrocyte ghost carriers must be characterized in biologically accurate disease models. The cecal ligation and puncture (CLP) model remains the most widely accepted and clinically representative murine model of polymicrobial sepsis, recapitulating the hemodynamic instability, cytokine storm, hepatic macrophage activation, and multi-organ dysfunction that characterize human abdominal sepsis [30,59]. Biodistribution studies conducted in CLP-induced septic mice—rather than in healthy or lipopolysaccharide-challenged animals—are essential for validating the hepatic accumulation and Kupffer cell uptake that the IACGT framework predicts. Furthermore, protein corona characterization must be performed in septic rather than healthy plasma, given the known compositional differences in acute-phase proteins and complement components under inflammatory conditions [64,65]. In vitro evaluation should include co-culture systems incorporating lipopolysaccharide-activated primary Kupffer cells and hepatocytes, as well as sinusoidal endothelial cell models under inflammatory stimulation, to generate mechanistically relevant uptake and cytotoxicity data prior to in vivo studies.

Stage 3—Payload Selection and Intracellular Release Engineering

The therapeutic benefit of IACGT-guided delivery depends critically on the selection of payloads that address the mechanistic drivers of septic liver injury and on release kinetics that match the dynamic inflammatory timeline of sepsis. Therapeutically rational payloads for Kupffer cell-targeted erythrocyte ghosts include the following: NF-κB pathway inhibitors such as BAY 11-7082 or parthenolide, which can attenuate the cytokine amplification loop driving hepatocellular injury [84,85]; mitochondria-targeted antioxidants such as MitoQ or SkQ1, which address the mitochondrial dysfunction central to hepatocyte death in sepsis [40]; nucleic acid-based payloads, including siRNA targeting TNF-α, IL-1β, or NLRP3, for which the macrophage cytosol represents the therapeutically relevant intracellular compartment [79,86]; and broad-spectrum antibiotics, such as ceftriaxone, that benefit from macrophage-mediated intracellular delivery in bacterial sepsis, as supported by existing erythrocyte ghost loading evidence [213]. Release kinetics should be engineered to exploit the lysosomal acidification that follows phagocytosis, using pH-responsive membrane compositions or fusogenic lipid components that enable endosomal escape and cytosolic payload delivery within the activated macrophage [87].

Stage 4—Manufacturing Feasibility and GMP Pathway

The clinical translation of erythrocyte ghost-based carriers will ultimately depend on the establishment of a scalable, reproducible, and GMP-compliant manufacturing process. From a process development perspective, hypotonic lysis-based ghost preparation is the most technically mature loading strategy and has been implemented at clinical scale by both Erytech Pharma and EryDel [214,215,216]. However, the preparation of ghosts from septic autologous erythrocytes introduces additional process challenges: oxidatively damaged membranes exhibit increased fragility and heterogeneous rupture kinetics under hypotonic conditions, potentially resulting in incomplete intracellular content removal and variable drug loading efficiency [206]. Quality control assays must therefore include membrane protein integrity profiling (particularly for CD47, band 3, and glycophorin A), phosphatidylserine surface quantification, deformability assessment by ektacytometry, and sterility and endotoxin testing—all conducted on the final drug-loaded product rather than on raw ghost preparations [217]. For allogeneic ghost platforms, the additional layer of donor blood group matching, minor antigen screening, and pathogen inactivation must be integrated into the GMP workflow, drawing on established transfusion medicine standards [218,219].

Stage 5—Regulatory Strategy

Erythrocyte ghost-based drug carriers occupy a regulatory space that intersects advanced therapy medicinal products (ATMPs), combination products, and biological medicines, depending on jurisdiction and the nature of the active payload. In the European Union, autologous cell-derived carriers loaded with a pharmacologically active substance are likely to be classified as somatic cell therapy medicinal products under the ATMP Regulation (EC No 1394/2007), requiring a centralized marketing authorization procedure through the European Medicines Agency [94]. In the United States, the FDA’s Center for Biologics Evaluation and Research (CBER) would likely exercise primary jurisdiction, with the product classified as a biological drug-device or biological combination product. Early engagement with regulatory agencies through pre-IND or scientific advice meetings is therefore a critical translational step that should be initiated in parallel with preclinical studies, not deferred to late-stage development. The regulatory experience accumulated through ERY-ASP and EryDex development provides a practical precedent that can be used to anticipate the clinical chemistry, manufacturing and controls (CMC) requirements, and the non-clinical safety package that regulators will expect [215,220].

## 9. Translational Barriers and Unmet Needs

Over the past decade, publications related to biomimetic cell membrane-coated nanocarriers have increased substantially, reflecting growing interest in biologically inspired drug delivery platforms. Within this expanding field, erythrocyte membrane-derived systems represent a significant proportion of blood cell-based biomimetic nanotechnology studies, largely driven by their immune compatibility and long circulation properties. However, qualitative assessment of the PubMed- and Scopus-indexed literature suggests that the majority of these studies are concentrated in oncology, systemic drug delivery, and immune evasion applications, while comparatively fewer investigations focus on organ-specific targeting in inflammatory diseases. In particular, studies directly evaluating erythrocyte ghost or erythrocyte membrane-coated systems for therapeutic liver targeting or sepsis-associated organ dysfunction remain relatively limited, indicating an important opportunity for further translational investigation.

Erythrocyte membrane-derived systems have demonstrated considerable promise in clinical settings, largely due to their favorable biocompatibility and prolonged circulation behavior. One of the most advanced examples is ERY-ASP, an erythrocyte encapsulated formulation of L-asparaginase developed by Erytech Pharma. By enclosing enzyme within erythrocytes, this system masks immunogenic epitopes, thereby extending the drug’s circulating half-life and reducing immune-mediated clearance. The intrinsic longevity of erythrocytes further supports sustained enzymatic activity, while gradual drug release minimizes the risk of acute toxicity associated with high systemic concentrations. ERY-ASP has been extensively evaluated in acute lymphoblastic leukemia (ALL). Phase II/III clinical trials (NCT01518517) reported a reduced incidence of hypersensitivity reactions and prolonged maintenance of therapeutic asparaginase activity [214]. In a subsequent phase II clinical trial (NCT03267030), the safety and pharmacological profile of ERY-ASP were evaluated involving ALL patients hypersensitive to PEG-asparaginase. The treatment was well tolerated, with the majority of patients showing asparaginase activity levels above the therapeutic target [215]. Unfortunately, Erytech Pharma has abandoned seeking approval for ERY-ASP for the treatment of hypersensitive ALL due to the changing competitive landscape [216]. Beyond hematologic malignancies, ERY-ASP was also explored in solid tumors. Although a phase II study in pancreatic cancer demonstrated improved progression-free survival when combined with chemotherapy, the subsequent phase III trial (NCT03665441) failed to meet its primary overall survival end-point [215]. Similarly, a phase II/III trial in triple-negative breast cancer (NCT03674242) was terminated after showing no clinical benefit, leading to the cessation of ERY-ASP development for these indications [216]. Furthermore, the Italian company (EryDel) have developed EryDex, a sustained-release dexamethasone delivery system, which demonstrated effective therapeutic results for ataxia-telangiectasia, a rare genetic disorder. Currently, the study is in its Phase II pilot trial (NCT01255358), where the product is undergoing pharmacokinetic studies in the U.S. and is awaiting a Phase III trial [220].

While erythrocyte-based systems offer clear advantages in biocompatibility and circulation longevity, clinical translation has remained limited due to persistent biological, logistical, and regulatory challenges. A long-standing concern relates to transfusion-transmitted infections. Historical outbreaks linked to contaminated blood products were largely the result of inadequate donor screening and insufficient processing. Advances in modern transfusion medicine, including stringent donor selection and pathogen screening protocols, have substantially reduced these risks. Nevertheless, emerging infectious diseases continue to challenge blood safety infrastructure [221,222]. The COVID-19 pandemic exposed vulnerabilities in donor availability and blood supply chains, underscoring that infection risk, while mitigated, remains an unresolved translational consideration for erythrocyte-derived therapeutics, particularly in acute conditions such as sepsis, where rapid deployment is essential.

Manufacturing and standardization present additional barriers. Allogeneic erythrocyte-based systems require careful donor sourcing, cold-chain storage, and batch-specific quality control. Variability in donor erythrocyte composition, membrane lipid content, and protein expression may influence biodistribution, immune recognition, and therapeutic payload stability. In contrast, autologous erythrocytes derived from septic patients may exhibit altered membrane composition, deformability, and surface signaling due to systemic inflammation, oxidative stress, and metabolic dysregulation. These disease-associated alterations can unpredictably affect carrier biodistribution, drug release kinetics, and safety, leading to batch-to-batch variability that complicates regulatory approval. Such biological heterogeneity poses a greater challenge than that encountered with chemically defined synthetic carriers [197,217]. Shelf-life limitations further constrain clinical feasibility. Isolated erythrocytes have a finite storage window, and drug-loaded erythrocyte formulations often require fresh preparation to preserve membrane integrity and functionality [19,223,224]. This requirement is poorly aligned with the realities of emergency and intensive care settings, where sepsis treatment demands ready-to-use formulations and rapid administration.

Targeting specificity remains another unresolved issue. The biodistribution of erythrocyte-based carriers is governed by a combination route of administration, loading strategy, and surface modification. Variations in injection site can markedly alter organ accumulation profiles. For instance, right jugular vein injection enhanced brain targeting efficiency while tail vein injection enriches liver/spleen accumulation [225]. Surface-bound payloads favor rapid release and phagocyte uptake, while intracellular encapsulation prolongs circulation but risks compromising native erythrocyte properties [187,226]. Membrane fusion and hybridization strategies offer additional control over targeting behavior, yet increase formulation complexity and regulatory burden [227,228,229]. Achieving reliable and disease-adaptive liver targeting therefore requires integrated optimization of carrier design, administration strategy, and pathological context—an approach that has not yet been standardized for septic liver injury.

Taken together, the unmet need in erythrocyte-mediated drug delivery is not the absence of biological rationale, but the lack of clinically adaptable designs that account for infection risk, biological variability, manufacturing constraints, and disease-specific pathophysiology. For sepsis-associated liver failure, future progress will depend on developing standardized, scalable, and inflammation-aware erythrocyte ghost platforms that align with the operational demands of critical care medicine. Addressing these translational barriers is essential for converting the inherent advantages of erythrocyte-based systems into tangible clinical benefit.

### Advancing Nanomedicine in Sepsis Toward Clinical Translation

The translational attrition rate in nanomedicine remains disproportionately high, with the large majority of preclinical candidates failing to advance beyond early-stage development [227]. In sepsis, this attrition is compounded by biological, logistical, and clinical trial design challenges that require explicit acknowledgement and proactive mitigation in the development strategy.

A practical barrier that is under-addressed in the nanomedicine literature is the heterogeneity of the patient population in whom these systems would be deployed. Sepsis is not a single disease entity but a syndrome encompassing diverse infectious sources, varying degrees of immune activation, different patterns of organ involvement, and highly variable individual pharmacokinetic profiles shaped by age, comorbidity, prior medications, and the specific causative pathogen [8,9]. Under these conditions, a delivery system tuned to a specific biological context—say, heightened Kupffer cell activity or complement activation which may not behave consistently across the broader population. It is therefore difficult to justify a “one-size-fits-all” approach.

A more realistic strategy would involve some form of patient stratification, introduced early rather than retrofitted later. Biomarkers could help here, although their predictive value in this setting is still evolving. Circulating soluble CD163, for instance, may reflect Kupffer cell activation; complement fragments such as C3a and C5a could indicate ongoing complement engagement; and cell-free hemoglobin might serve as a proxy for erythrocyte membrane stress [204,206,208]. These markers are already measurable in clinical laboratories, which makes them practical candidates for enrichment strategies in early-phase trials. Still, it is worth noting that their specificity—and how well they map onto delivery system performance—remains to be fully clarified.

Clinical trial design presents a separate but related challenge. Conventional phase II/III studies in sepsis often rely on endpoints such as 28-day mortality. In practice, these trials are frequently underpowered. Mortality rates vary widely across centers and patient subgroups, and confounding factors are difficult to control [4,30]. As a result, even interventions with biological activity may fail to show statistical benefit. Adaptive trial designs offer a possible way forward. By incorporating interim analyses, biomarker-guided enrolment, and pharmacodynamic readouts, these designs may provide a clearer signal of whether a targeted delivery system is doing what it is intended to do. Endpoints such as hepatic cytokine output, liver injury markers (ALT, bilirubin, factor V), or even intrahepatic drug levels measured by microdialysis could be more informative at the proof-of-concept stage. These methodological considerations should be incorporated into the translational development plan from the earliest stages rather than deferred to clinical phase design.

Finally, the operational context of intensive care medicine imposes practical constraints that must be reflected in formulation design. Intravenous drug products intended for use in critically ill patients must be compatible with common co-medications, stable over the temperature fluctuations encountered in clinical storage and transport, administrable through central venous catheters without aggregation or membrane adsorption, and manufacturable in dosage forms that do not require preparation by highly trained personnel under time pressure [9,61]. These constraints argue for lyophilized or cryopreserved erythrocyte ghost formulations that can be reconstituted at the bedside, analogous to the presentation formats used for approved erythrocyte-encapsulated enzyme products [223,224]. Shelf-life extension through optimized cryoprotectant formulations, which builds on established RBC cryopreservation protocols using glycerol-based systems validated in transfusion medicine [230], represents a technically feasible near-term priority for advancing erythrocyte ghost systems toward clinical readiness.

## 10. Erythrocyte Membrane Sourcing Strategies in Sepsis from Autologous and Allogeneic Approaches

In septic liver failure, immunological precision and manufacturing urgency collide. The source of the erythrocyte membrane becomes not a technical detail, but a determinant of safety, scalability, and clinical viability. The choice between autologous and donor-derived (allogeneic) RBC ghosts introduces distinct challenges.

Autologous RBC ghosts offer the clearest immunological advantage; as they originate from the same individual, risks of alloimmune reactions, hemolytic transfusion responses, and minor antigen incompatibilities are minimized [109,231,232]. This is particularly relevant in critically ill patients, where immune dysregulation already complicates outcomes. The logistics of rapid, sterile, point-of-care membrane preparation under septic conditions further complicate implementation. Time-sensitive manufacturing, endotoxin control, and GMP-compliant processing in an intensive care setting represent substantial operational challenges.

Allogeneic RBC ghosts, by contrast, offer structural consistency and scalability. Donor erythrocytes are typically collected from healthy individuals under standardized blood banking conditions, ensuring membrane integrity and quality control [218]. Batch production enables characterization of membrane protein composition, lipid stability, deformability, and sterility prior to clinical use [219]. This improves reproducibility and facilitates regulatory standardization. In emergency contexts like septic shock, off-the-shelf availability is a clear logistical advantage. Yet allogeneic systems introduce immunological and regulatory complexity. Even when major ABO and Rh matching is ensured, minor blood group antigens may provoke alloimmunization [233]. There is also the theoretical risk of pathogen transmission, although modern blood screening significantly reduces this concern [234]. Cryopreserved RBCs have been shown to retain structural integrity for extended periods when stored under appropriate glycerol-based protocols [230]. Preventive autologous erythrocyte biobanking could reconcile immunological safety with manufacturing readiness. It transforms autologous RBC ghost production from an emergency bedside procedure into a preemptive, standardized resource embedded within transfusion infrastructure. While ambitious, such a strategy aligns with principles of precision medicine and may become feasible as cell-based therapeutic platforms.

The choice between autologous and allogeneic RBC ghosts should not be framed as binary. Instead, it represents a spectrum of immunological safety, biological integrity, scalability, and regulatory complexity. In an acute septic setting, off-the-shelf allogeneic systems may provide immediate feasibility. Meanwhile, for personalized or repeated administrations, bio-banked autologous membranes may offer superior immunological compatibility. Ultimately, translational success will depend on integrating membrane biology, manufacturing science, and transfusion medicine infrastructure.

## 11. Conclusions and Future Directions

The convergence of sepsis biology and biomimetic drug delivery science at the juncture examined in this review reveals a field at an inflection point. The rationale for erythrocyte ghost-based liver targeting in abdominal sepsis is compelling and mechanistically grounded: the disease itself amplifies the very clearance pathways through which these carriers engage the hepatic immune microenvironment. Yet the gap between biological rationale and clinical reality remains wide, and bridging it requires more than incremental refinement of carrier design. It requires a fundamental reorientation of how delivery systems for acute inflammatory organ failure are conceptualized, evaluated, and developed.

The IACGT framework proposed in this review represents one such reorientation. By positioning inflammation-driven Kupffer cell clearance as a targeting mechanism rather than a pharmacokinetic obstacle, it challenges the stealth-first paradigm that has dominated nanocarrier development for two decades and offers a disease-aligned conceptual alternative grounded in septic pathophysiology. This framework is not merely theoretical—it generates specific, testable design predictions: that complement-modulating surface chemistries will outperform PEG-based stealth coatings in septic models; that phosphatidylserine-exposing ghost preparations will exhibit superior Kupffer cell uptake compared to PS-masked counterparts in CLP animals; and that CD47 preservation alone will be insufficient to regulate macrophage engagement in the inflammatory plasma environment of sepsis. These predictions constitute a translational research agenda that can be systematically addressed in appropriately designed preclinical studies.

Looking forward, several converging technological developments hold particular potential for advancing this field. The maturation of microfluidic ghost preparation platforms offers the prospect of continuous-flow, GMP-compatible erythrocyte ghost production with tight control over membrane protein orientation, drug loading uniformity, and particle size distribution—addressing the batch-to-batch variability that has historically limited reproducibility in erythrocyte-based formulations [110,235,236]. Advances in single-cell proteomics and spatial transcriptomics of the septic liver are generating an increasingly granular understanding of Kupffer cell heterogeneity, macrophage zonation, and sinusoidal immune dynamics that can inform the next generation of carrier surface engineering with unprecedented biological specificity [198,199]. The integration of physics-informed machine learning models with high-throughput nanoparticle screening platforms is beginning to enable computational prediction of carrier-macrophage interactions under inflammatory conditions, offering a route to rational optimization of IACGT-compatible surface compositions before costly in vivo studies are initiated [83].

The preventive autologous biobanking strategy represents the longest-horizon translational concept in this review but also arguably the most consequential. If erythrocyte ghost platforms are to achieve the immunological precision that autologous systems offer without the manufacturing constraints imposed by acute illness, the solution must be embedded in preventive infrastructure rather than reactive bedside production. The technical feasibility of long-term erythrocyte cryopreservation is established [230,237]; what remains to be developed is the institutional framework—covering consent, storage, quality control, and on-demand processing—that would integrate such biobanking within existing transfusion medicine systems. Pilot studies evaluating membrane protein stability, ghost fabrication efficiency, and drug loading capacity after extended cryopreservation of healthy donor erythrocytes would represent a tractable near-term research priority with high translational value.

Ultimately, the clinical translation of biomimetic erythrocyte ghost platforms for septic liver failure will depend on the convergence of three disciplines that rarely communicate in current research practice: nanomedicine, transfusion medicine, and critical care pharmacology. Each brings indispensable expertise, nanomedicine in carrier engineering and biological characterization; transfusion medicine in erythrocyte biology, donor management, and GMP cell processing; and critical care in clinical trial design, patient stratification, and the operational realities of intensive medicine. Building research consortia and translational programs that genuinely integrate these perspectives—rather than treating nanomedicine as the primary discipline and the others as technical service providers—is the organizational prerequisite for meaningful progress. The patients who stand to benefit from more effective, targeted therapy for septic organ failure deserve no less than this level of scientific and institutional ambition.

## Figures and Tables

**Table 1:** Comparison of cell membrane-coated nanoparticle systems for biomimetic drug delivery.

Membrane Type	Principle	Functions	Advantages	Limitations	References
Red Blood Cell (RBC) Membrane-Coated NPs	Nanoparticles are cloaked with RBC membrane vesicles to present “self” surface markers that reduce immune recognition and prolong circulation in blood.	Immune evasion and extended systemic circulation; improved tumor accumulation; reduced RES clearance.	Prolonged blood circulation; enhanced stability and biocompatibility; reduced immune clearance.	Poor innate targeting without additional modifications; fabrication challenges for uniform coating; variable in vivo targeting efficiency.	[127,128,129]
Macrophage Membrane-Coated NPs	Synthetic nanocarriers are decorated with macrophage membranes to inherit homing ability to inflammatory or tumor tissues and to evade immune detection.	Targeted tumor delivery; pH-responsive drug release; immune system interaction; tumor microenvironment homing.	Enhanced tumor targeting and penetration; immune evasion; controlled drug release in response to TME.	Complex membrane extraction; stability of membrane during circulation; potential off-target immune interactions.	[130,131,132]
Cancer Cell Membrane-Coated NPs (CCMNPs)	Nanoparticles cloaked with cancer cells membranes maintain surface antigens and adhesion molecules that facilitate homotypic targeting (tumor cells recognize each other).	Tumor homing via self-recognition; immune escape via CD47; tumor antigen presentation.	Homotypic tumor targeting; immune evasion; potential antigen presentation for immunotherapy.	Requires culturing and membrane extraction from cancer cells; risk of immunogenicity; scalability issues.	[133,134,135]
Hybrid/Multi-Membrane Coated NPs (e.g., RBC + Platelet)	Membrane fusion from two or more cell types to combine functional surface properties (e.g., long circulation + targeting).	Combined stealth + targeting functions; customizable surface bioactivity.	Multi-functionality; synergistic performance (immune evasion + targeting).	Very complex fabrication; reproducibility issues; regulatory hurdles.	[136,137]
Immune Cell Membrane (e.g., WBC) Coated NPs	Membranes from white blood cells convey receptor profiles that naturally home to inflammatory sites and aid immune system interactions.	Target inflammation and tumor microenvironment; enhanced extravasation and site-specific uptake.	Improved targeting to inflamed/tumor tissues and evasion of opsonization.	Difficult extraction coupled with complex vesicle stability; inconsistent membrane composition.	[138,139]
Platelet Membrane-Coated NPs	Platelet surface proteins (e.g., P-selectin) give NPs ability to interact with damaged vasculature and pathogens.	Targeting to injured blood vessels; cancer site homing; bacterial binding.	Immune evasion with targeting to injury/inflammation; native adhesion properties.	Complex membrane isolation; potential off-target adhesion; and scalable challenges.	[140,141]
Cell Membrane-Coated NPs	Uses stem cell membranes with innate tissue-tropism and regenerative signaling molecules.	Target regenerative sites; deliver therapeutic cargos to injured tissue.	Natural homing ability; potential multi-disease targeting.	Limited clinical data; complex isolation and characterization.	[142,143]

**Table 2:** Preparation-derived limitations of erythrocyte ghost-based carriers and their in vivo drug delivery consequences in the context of sepsis-induced liver failure.

Preparation-Derived Limitation	In Vivo Delivery Consequence	References
Variable encapsulation efficiency	Inconsistent hepatic drug dose results in unpredictable pharmacokinetics and therapeutic effect; narrow therapeutic windows in septic patients may increase the risk of under- or over-dosing.	[184,188]
Incomplete membrane resealing	Premature systemic release of encapsulated payload before carrier reaches the liver may lead to off-target exposure and loss of localization advantage.	[189,190]
Loss or alteration of surface proteins, including CD47	Impaired CD47-SIRPα inhibitory signaling reduces circulation time and shifts biodistribution toward the spleen, reducing effective hepatic targeting.	[191,192]
Payload–membrane physicochemical interactions	Lipophilic drugs may diffuse out before macrophage uptake; hydrophilic drugs may be trapped until late degradation, resulting in mismatch between intended and actual release site.	[185,193]
Sepsis-induced oxidative membrane damage	Oxidative injury to septic erythrocyte membranes increases fragility and heterogeneous rupture kinetics, resulting in reduced and heterogeneous drug loading, incomplete resealing, and accelerated monocyte-mediated clearance.	[194,195]
Inflammation-induced alterations in hepatic receptor expression	Dynamic changes in hepatic receptor expression and clearance pathways under systemic inflammation may redirect carriers away from intended liver compartments, undermining targeting specificity.	[196,197]

## Data Availability

No new data were created or analyzed in this study. Data sharing is not applicable to this article.

## Abbreviations

The following abbreviations are used in this manuscript:

AAD	Anti-angiogenic drug
ALL	Acute lymphoblastic leukemia
ApoE	Apolipoprotein E
ASGPR	Asialoglycoprotein receptor
AuNRs	Gold nanorods
BBB	Blood–brain barrier
bFGF	Basic fibroblast growth factor
CD47	Cluster of differentiation 47
CCM-NPs	Cancer cell membrane-coated nanoparticles
CNPs	Cell membrane-coated nanoparticles
DAMPs	Damage-associated molecular patterns
EVs	Extracellular vesicles
Fc	Fragment crystallizable region
FDG	Fluorodeoxyglucose
GMP	Good manufacturing practice
IACGT	Inflammation-adaptive clearance-guided targeting
IL	Interleukin
IL-1β	Interleukin-1 beta
IL-4	Interleukin-4
IL-5	Interleukin-5
IL-6	Interleukin-6
IL-13	Interleukin-13
KO-RDNVs	Knockout red blood cell-derived nanovesicles
LDL	Low-density lipoprotein
LPS	Lipopolysaccharide
LSECs	Liver sinusoidal endothelial cells
MHC	Major histocompatibility complex
MODS	Multiple organ dysfunction syndrome
MSNs	Mesoporous silica nanoparticles
NF-κB	Nuclear factor kappa B
NK cells	Natural killer cells
NLRs	Nucleotide-binding oligomerization domain-like receptors
NOD	Nucleotide-binding oligomerization domain
NPs	Nanoparticles
PAMPs	Pathogen-associated molecular patterns
PD-L1	Programmed death-ligand 1
PEG	Polyethylene glycol
PNPs	Platelet membrane-coated nanoparticles
PS	Phosphatidylserine
RBCs	Red blood cells
RBC-coated NPs	Red blood cell membrane-coated nanoparticles
RDNVs	Red blood cell-derived nanovesicles
RES	Reticuloendothelial system
ROS	Reactive oxygen species
siRNA	Small interfering RNA
SIRPα	Signal regulatory protein alpha
TLRs	Toll-like receptors
TNF-α	Tumor necrosis factor alpha
VDA	Vascular disruption agent
WBCs	White blood cells
WT-RDNVs	Wild-type red blood cell-derived nanovesicles
